# Genome-wide SNP analysis of *Plasmodium falciparum* shows differentiation at drug-resistance-associated loci among malaria transmission settings in southern Mali

**DOI:** 10.3389/fgene.2022.943445

**Published:** 2022-10-04

**Authors:** Aoua Coulibaly, Mouhamadou Fadel Diop, Aminatou Kone, Antoine Dara, Amed Ouattara, Nicola Mulder, Olivo Miotto, Mahamadou Diakite, Abdoulaye Djimde, Alfred Amambua-Ngwa

**Affiliations:** ^1^ Malaria Research and Training Center, University of Science, Techniques, and Technologies of Bamako, Bamako, Mali; ^2^ Computational Biology Division, University of Cape Town, Cape Town, South Africa; ^3^ Disease Control and Elimination, Medical Research Council Unit The Gambia at LSHTM, Banjul, Gambia; ^4^ University of Maryland Baltimore, Baltimore, MD, United States; ^5^ Mahidol Oxford Tropical Medicine Research Unit, Mahidol University, Bangkok, Thailand

**Keywords:** malaria, drug resistance, genetic variation, positive selection, differentiation

## Abstract

*Plasmodium falciparum* malaria cases in Africa represent over 90% of the global burden with Mali being amongst the 11 highest burden countries that account for 70% of this annual incidence. The persistence of *P. falciparum* despite massive global interventions is because of its genetic diversity that drives its ability to adapt to environmental changes, develop resistance to drugs, and evade the host immune system. Knowledge on *P. falciparum* genetic diversity across populations and intervention landscape is thus critical for the implementation of new strategies to eliminate malaria. This study assessed genetic variation with 12,177 high-quality SNPs from 830 Malian *P. falciparum* isolates collected between 2007 and 2017 from seven locations. The complexity of infections remained high, varied between sites, and showed a trend toward overall decreasing complexity over the decade. Though there was no significant substructure, allele frequencies varied geographically, partly driven by temporal variance in sampling, particularly for drug resistance and antigen loci. Thirty-two mutations in known drug resistance markers (*pfcrt*, *pfdhps*, *pfdhfr*, *pfmdr1*, *pfmdr2,* and *pfk13*) attained a frequency of at least 2% in the populations. SNPs within and around the major markers of resistance to quinolines (*pfmdr1* and *pfcrt*) and antifolates (*pfdhfr* and *pfdhps*) varied temporally and geographically, with strong linkage disequilibrium and signatures of directional selection in the genome. These geo-temporal populations also differentiated at alleles in immune-related loci, including, *protein E140*, *pfsurfin8*, *pfclag8*, and *pfceltos*, as well as *pftrap*, which showed signatures of haplotype differentiation between populations*.* Several regions across the genomes, including five known drug resistance loci, showed signatures of differential positive selection. These results suggest that drugs and immune pressure are dominant selective forces against *P. falciparum* in Mali, but their effect on the parasite genome varies temporally and spatially. Interventions interacting with these genomic variants need to be routinely evaluated as malaria elimination strategies are implemented.

## Introduction

Human malaria caused by infection with *Plasmodium falciparum* is one of the most deadly parasitic diseases, particularly in Africa, where fatalities have been exacerbated by interruptions in interventions due to the COVID-19 pandemic (Dyer, 2020). According to the 2021 World Health Organization (WHO) Malaria Report, there were 241 million cases of malaria in 2020 and 627,000 deaths worldwide (World Health Organisation, 2021). Most of these cases occurred in the African region, primarily among women and children under the age of five. To mitigate these, current malaria intervention strategies include early diagnosis with rapid diagnostic tests (RDTs), treatment of clinical cases with artemisinin combination therapies (ACTs), intermittent preventive treatment for pregnant women (IPTp) with sulfadoxine–pyrimethamine (SP); seasonal malaria chemoprevention (SMC) in children with SP and amodiaquine, indoor residual spraying (IRS) with insecticides, and use of long-lasting insecticide-treated bed nets (LLINs) against the vectors (Hemingway et al., 2016). Despite the decline in malaria incidence from long-term and intensive implementation of these interventions, malaria remains highly endemic across Africa, including Mali. The entire population of Mali is at risk of malaria, although transmission varies across the country’s five geo-climatic zones (Cissoko et al., 2022). Transmission remains high throughout most of the year in the central and southern regions, where more than 90 percent of the population lives, but it is seasonal and epidemic in the desertic North (PMI, 2020). In 2020 alone, Mali officially recorded 7.2 million cases of malaria and 19,300 estimated deaths for a population of about 20 million (World Health Organisation, 2021).

As is the case in most endemic regions, the main intervention against malaria morbidity and mortality has been drugs, with the accompanying evolution of drug resistance that led to changes in first-line treatment from cheap drugs such as chloroquine to the current ACTs. Chloroquine was used in Mali as first-line treatment for malaria for more than 50 years, leading to the development of resistance in *P. falciparum* (Wellems and Plowe, 2001). Chloroquine resistance has been associated with genetic mutations in the chloroquine resistance transporter (*pfcrt*) and multidrug resistance 1 (*pfmdr1*) genes, which spread through Africa from Southeast Asia, leaving strong signatures of selective sweeps in the genome of *P. falciparum* (Moran and Bernard, 1989; Djimde et al., 2001). Similarly, mutations and selective sweeps emerged around dihydrofolate reductase (*pfdhfr*) and dihydropteroate synthetase genes (*pfdhps*), which are associated with SP resistance, combination drugs that were introduced to contain chloroquine resistance (Pearce et al., 2009; Mita, 2010). In fact, *P. falciparum* has developed resistance to most previous drugs and current recommended ACTs combine a fast acting artemisinin derivative (ART) and a long-lasting partner drug such as lumefantrine, piperaquine, mefloquine, or amodiaquine, to limit resistance development (World Health Organisation, 2006). Although ACTs have been highly effective in reducing the morbidity and mortality associated with malaria, artemisinin resistance has already been confirmed in five countries of the Greater Mekong Sub-region (GMS) including Cambodia, the Lao People’s Democratic Republic, Myanmar, Thailand, and Vietnam (Dondorp et al., 2009). In these countries, resistance to ARTs has been associated with single nucleotide polymorphisms (SNPs) in the propeller domain of the *P. falciparum* kelch-13 (*pfk13*) gene on chromosome 13 (Ariey et al., 2014). These polymorphisms are largely absent in Africa and have not yet been described in Mali (Ndwiga et al., 2021). However, there are fears that they could emerge independently in the future or be introduced from Asia. These will add to the already existing resistance to previous monotherapies, some of which are partner drugs in ACTs. These drug resistance polymorphisms, therefore, need to be rigorously monitored.

Genomic polymorphisms in *P. falciparum* populations not only enable drug resistance but also allow for adaptation of this malaria parasite to other environmental changes, including evasion of host immune defenses across several tissues (Molina-Cruz et al., 2015; Gomes et al., 2016). Interaction with immune mechanisms and adaptation also leave signatures of selection in the genome around specific antigens that could be targeted for development of future immune interventions. Genetic variation in these targets could affect their broad efficacy across endemic regions as has been reported for the only available malaria vaccine, RTS,S, now approved for global roll-out (Neafsey et al., 2015). Therefore, characterizing population genetic diversity and understanding the main drivers of genetic variation and differentiation would facilitate the design of new antimalarial tools and refine implementation strategies to reduce malaria transmission, morbidity, and mortality. Already, differences in *P. falciparum* genetic diversity and the extent of adaptative signatures in genomes from populations with differences in malaria transmission intensities in Africa have been described (Amambua-Ngwa et al., 2019). These differences may also be imposed by differences in the history of interventions, human host ethnicity, and diversity in vector populations. How these have affected genomic variation and the dynamics of markers of adaptation can be further elucidated by focusing on smaller population units as shown in Mauritania, which borders Mali to the west of West Africa (Duffy et al., 2018). Such high-resolution analysis of populations could also resolve genetic differences in *P. falciparum* that may be responsible for differences in virulence and immunity, vital information for the optimal design of malaria vaccines (Engelbrecht et al., 1995).

The overall goal of this study was, therefore, to determine *P. falciparum* genomic variation across malaria endemic settings in southern Mali, where transmission is generally high and multiclonal infections are common, but differences in the environment and local host populations could affect parasite populations differently. Classical and local signatures of positive and balancing selection are reported, showing geographic and temporal differentiation around loci associated with drug resistance and immunity, despite the uniformity in national malaria intervention strategies over the decade of sampling.

## Methods

The original study was approved by the Ethics Committee of the Faculty of Pharmacy and the Faculty of Medicine and Odonto-Stomatology of USTTB, Bamako, Mali and each study participant provided a signed informed consent.

### Study sites

**FIGURE 1:**
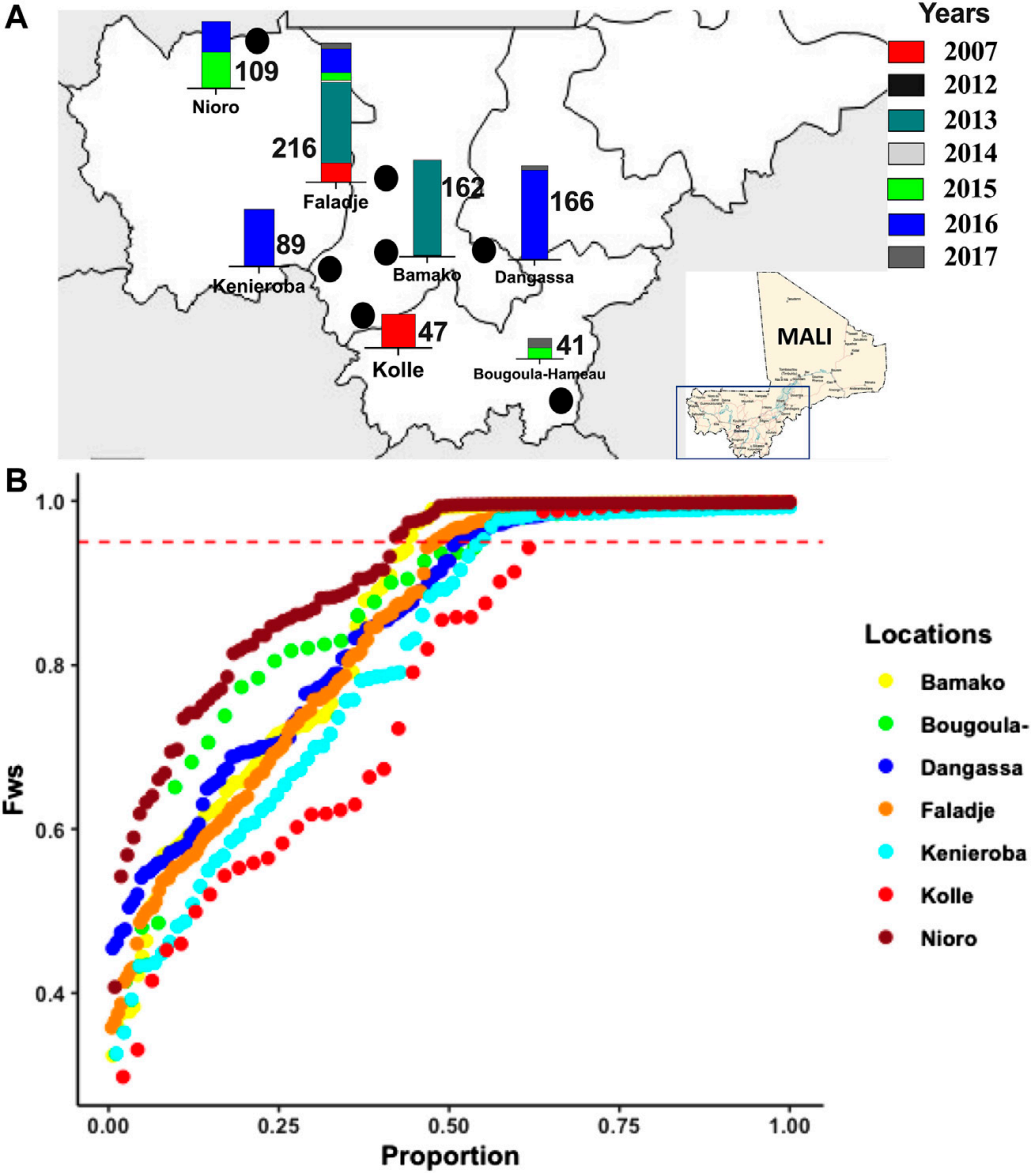
Geographic and temporal malaria parasites sampled across different endemic sites in Mali between 2007 and 2017, showing, **(A)** sampling locations in Mali (marked with black points), and bars of number of samples per location from different years as color-coded. **(B)** Distribution of the *F*_WS_ inbreeding coefficient for infection complexity determined for isolates from different locations sampled.

Human malaria parasite infected blood samples were collected between 2007 and 2017 from seven malaria-endemic sites in southern Mali (Figure 1A, Supplementary Table S1), where the high malaria transmission season overlaps with the annual rainy season that occurs from June to December. Given the heterogeneity in years of sampling between sites, we refer to these sampling sites generally as “geo-temporal” populations. The sites included, Bamako, the capital of Mali, which is in the Sahelian zone and experiences relatively lower levels of malaria transmission due to its urban environment. The city has a shorter transmission season with a seasonal peak from June to November. The samples (162) analyzed from here were collected mostly in 2013. Three samples were from 2012.

Bougoula-Hameau (Bougoula) is a village in the suburban area of Sikasso, which is located 375 km southeast of Bamako. Malaria transmission is seasonal and mainly occurs from May to September (Fofana et al., 2022). The samples from this site were collected in 2015 and 2017.

Dangassa is in the south savannah grassland of eastern Mali, along the Niger River and has year-round access to fresh water. Malaria transmission in the area is both seasonal and perennial with parasite prevalence ranging from 40 to 50% of the population in the dry season (October–May) and 70–85% in the rainy season (June–September) (Konate et al., 2020). Most of the samples from here were from 2016 (158), with eight from 2017.

Faladje village is located 80 km northwest of Bamako with malaria transmission being both seasonal and perennial. Prevalence of parasitemia ranges from 40 to 50% of the population in the dry season (October–April) and 70–85% in the rainy season (May–September) (Cissoko et al., 2022). The samples (216) spanned the decade from 2007 to 2017, with the largest number (125) from 2013.

Kenieroba is a village of about 2,000 people and is located approximately 75 km southwest of Bamako (Cissoko et al., 2022). The 89 samples from this site were all collected in 2016.

Kolle is a rural village of 2,500 people located 55 km southeast of Bamako. Prevalence of parasitemia also ranges from 40 to 50% of the population in the dry season (October–May) and 70–85% in the rainy season (June–September) (Cissoko et al., 2022). This site presented the earliest sample set (Sangho et al., 2021) collected in 2007.

Finally, Nioro-du-Sahel (Nioro) is in the Sahelian region of Mali near the Islamic Republic of Mauritania with desert climate, limited rainfall, and the lowest malaria transmission (Hannah Keonker and Bouare, 2020). The samples from Nioro (109) were collected in 2015 and 2016.

### Data collection and pre-processing

From each participant, a dried blood spot (DBS) sample was collected by spotting whole blood obtained by finger prick onto filter paper cards. The collection was carried out in accordance with the SPOTMalaria protocol (www.malariagen.net/projects/SPOTMalaria), a project led by the Malaria Genetic Epidemiology Network (MalariaGEN) to harness genomic technologies in monitoring the evolution of malaria parasites. The filter papers were subjected to DNA extraction using the Qiagen DNA extraction protocol and the genome of *P. falciparum* in each sample was sequenced at the Wellcome Sanger Institute as previously described (MalariaGen et al., 2021). The short sequence read (fastq) files were mapped to the *P. falciparum* 3D7 reference genome with BWA-MEM and the resultant BAM files were used in variant calling following GATK best practice custom pipelines by the MalariaGEN *P. falciparum* community and Pf3K projects (https://www.malariagen.net/parasite/pf3k). For downstream analysis, vcf files of variants were filtered with *vcftools* (vcftools.github.io) to retain only biallelic coding SNPs supported by at least five reads and with a mapping quality greater than 30 (Supplementary Figure S1). Using R scripts, we iterated through filtration steps to remove SNPs or samples with excessive missingness and retained only SNPs genotyped for at least 80% of individuals and individuals with less than 20% of missing SNP calls. We then retained only high-quality PASS bi-allelic SNPs with a minor allele frequency of at least 0.02 (2%). The final dataset included 12,177 SNPs in 830 samples from an original set of 3,844,304 variants and 1270 samples (Supplementary Table S2).

### Population diversity, structure, and linkage disequilibrium

For each sample, within-host genetic diversity was assessed using the within-sample inbreeding coefficient (*F*_WS_ index) as described previously (Manske et al., 2012). *F*_WS_ was calculated using the *moimix* package implemented in R (Lee and Melanie, 2016). Pairwise SNP linkage disequilibrium (LD) represented by the correlation (*r* ^2^) between alleles at physically separated loci was calculated using PLINK version 1.92.0 for each chromosome per population. The *geom_smooth*() function from *ggplot2* was used to fit the distribution of *r* ^2^ for SNPs within a physical distance of 50 Kb.

To determine the potential structure between the geo-temporal populations, dimension reduction methods were used on a pruned set of 5541 SNPs with pairwise LD (*r* ^2^) of not greater than 0.02 between any pair of SNPs. The samples were clustered by principal component analysis (PCA) and multidimensional scaling analysis (MDS). PCA was performed using the *findclusters*() and *dapc*() within the R *adegenet* package. PCA clustering was also evaluated with snpRelate *snpgdsPCA*() function, which calculates the genetic covariance matrix from genotypes and computes the correlation coefficients between sample loadings and genotypes for each SNP. For MDS, pairwise identity by state (IBS) distance was generated in PLINK, followed by MDS in R using the *cmdscale*() function.

### Ancestry analysis

Ancestry was inferred using *tess3r*, which combines distance matrix factorization and spatial statistical methods to estimate ancestry coefficients (Caye et al., 2016). The optimal number of ancestries was determined from 10 runs of each K from K = 1 to K = 10. The root-mean-squared errors computed on each subset of loci were then used for cross-validation. The best K value was identified at the inflection point of increase in the cross-validation curve. Tess3r ancestry coefficients (q-matrix) were extracted for the optimal K and visualized using the *pophelper* package in R (Francis, 2017).

### Population differentiation

Overall genetic differentiation between geo-temporal population pairs was evaluated by *F*_ST_ using a Bayesian approach as applicable to populations with high geneflow, implemented by the *finepop* package in R (Kitada et al., 2017), following the Nei method as applied in the *mmod* package in R (Winter 2012). *Finepop* and *mmod* also estimated the Jost’s D, between populations. To determine differentiation per SNP, D, Gst, and Gstprime between all geo-temporal populations and temporal populations from Faladje were determined with *mmod*. Allele frequency differences between pairs of temporal populations for each SNP were also determined as a variant of *F*_ST_ (*pF*_ST_) as applied in *vclib*. Values of indices for SNPs were visualized using Manhattan plots. SNPs with values beyond the 99.5 percentile were further analyzed as outliers.

SNPs in the following drug resistance genes were extracted; chloroquine resistance transporter (*Pfcrt*), dihydrofolate reductase (*Pfdhfr*), dihydropteroate synthase (*Pfdhps*), kelch 13 (*Pfk13*), multidrug-resistance protein 1 (*Pfmdr1*), multidrug-resistance 2 (*Pfmdr2*), and plasmepsin II (*Pfpm2*). The frequencies of SNPs in these antimalarial drug resistance markers were calculated for each temporal or geo-temporal population using *vcftools*. Linkage disequilibrium between the SNPs was calculated and visualized using the *LDheatmap* R package (https://sfustatgen.github.io/LDheatmap/).

### Signatures of selection

To test if variation within genes deviated from neutrality, we calculated Tajima’s D for each gene with at least three SNPs per geo-temporal population using the *PopGenome* package in R (Pfeifer et al., 2014). Genes with a Tajima’s D value of at least 3 in two or more geo-temporal populations were considered as candidate targets for balancing selection. To determine genomic regions that might be under recent positive selection, we calculated the integrated haplotype homozygosity score (|iHS|), and the cross-population ratio of extended haplotype homozygosity (EHH) expressed as Rsb and XP-EHH for each SNP with the R package *REHH* (Gautier and Vitalis, 2012). For the iHS significance, the *REHH* package generates a two-sided *p*-value as −log10(2Φ (- |iHS|)), where *Φ* (- |iHS|)) represents the Gaussian cumulative distribution function. The generated *p* values were adjusted for multiple testing following the method of Benjamini, Hochberg, and Yekutieli to control for the false discovery rate (fdr), as implemented by the *p.adjust*() function in base R. SNP loci with fdr adjusted -log10 *p*-values (q-values) over the 99.5 percentile were considered as outliers and marking selective sweep regions.

### Statistical analysis

All statistical tests were conducted in R statistics software. To compare the differences in distribution of population genetic indices between geo-temporal populations, the Kruskal–Wallis non-parametric test was applied. For correlation between indices such as allele frequencies, Pearson’s correlation coefficients were derived using the R base *cor*() function. False discovery rate adjustment for multiple testing was applied to summary statistics of selection using the *p.adjust*() function in base R. Outlier SNPs or genes were selected by applying a 99.5 percentile to distribution of indices, retaining the top 0.5% of SNP loci for further characterization using PlasmoDB. Analysis of gene ontology enrichment for the biological function of selected genic loci was performed in PlasmoDB with a *p*-value cut-off of 0.05 for significance. Further clustering and visualization of ontology terms were performed using REVIGO (Supek et al., 2011). The codes used in analysis and figures shown in this study can be found at https://github.com/MPB-mrcg/Pfalciparum_Mali_geo_temp_popgen. Generic codes for statistical calculations and plots followed the manuals for the various packages and function implemented in R.

## Results

### High complexity of infection, low inter-SNP linkage disequilibrium, and lack of spatial substructure between populations

Within-host complexity of infection determined by the *F*_WS_ fixation index (inbreeding coefficient within a population), ranged from 0.32 (most polygenomic infections) to 1.0 (monogenomic infection). The distribution of *F*_WS_ was different across geo-temporal populations (Kruskal–Wallis, p = 1e-06), (Supplementary Figure S2A). The lowest mean *F*_WS_ was 0.78 for the 2007 isolates from Kolle and the highest was 0.91 in Nioro which is the site with the lowest reported malaria transmission and bordering Mauritania. The overall proportion of isolates with *F*_WS_ scores above 0.95, indicating a predominance of single genotype in the infection, was 0.56. This proportion was similar across all populations (*p*-value = 0.1949), except between Nioro (0.59) and Kolle (0.39), where the difference was significant (*p*-value = 0.03012), (Figure 1B). Classified by year of sampling, complex (polygenomic) infections remained high (Supplementary Figure S2B), though there was a small but significant increase in less complex infections in recent samples, with overall median *F*_WS_ increasing from 0.82 in 2007 to 0.88 in 2017 (Supplementary Figure S2C). Considering Faladje alone, which had samples across all years, *F*_WS_ fluctuated but the highest median (0.92) was for samples collected in 2017. The overall LD (*r* ^2^) between SNPs per population did not align with differences in complexity, as LD for all geo-temporal populations decayed rapidly below 0.05 within 5000 base pairs (Supplementary Figure S3).

**FIGURE 2:**
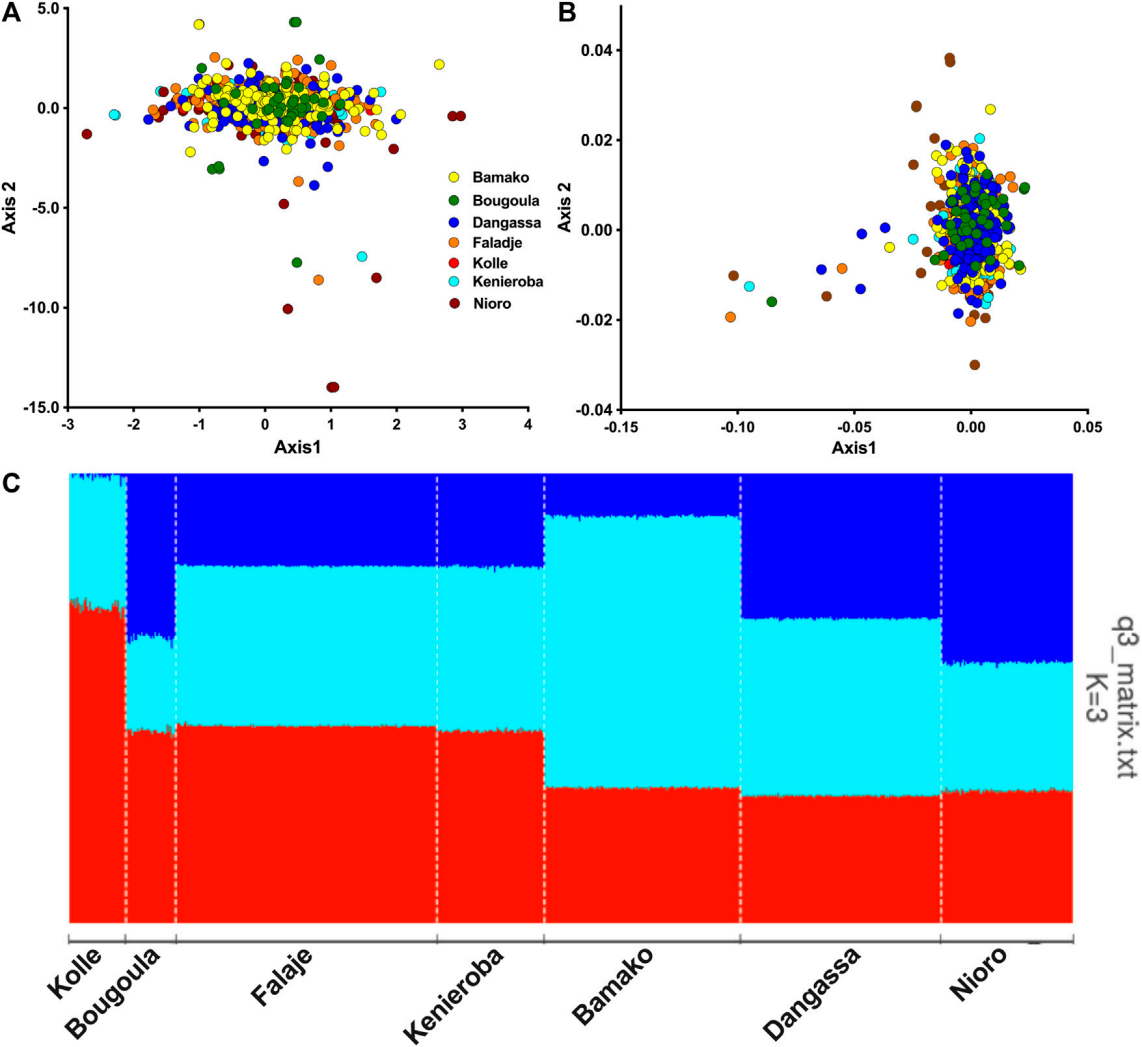
Population structure and ancestry of *P. falciparum* from Mali. **(A)** Plot of the first two components (Axis1 *vs*. Axis2) of principal component analysis (PCA) using genotype data from samples in Mali. **(B)** Scatter plot of first two dimensions of multidimensional scaling (MDS) of identity by state between pairs of isolates. Each point represents an isolate, color-coded by sites of sampling. **(C)** Barplot of ancestry proportions per sample and site of collection shown on the *x*-axis. Each ancestral population is indicated by a different color, (K1 (red), K2 (turquoise), and K3(blue)) and each vertical bar represents a single sample, colored according to the estimated ancestry proportions.

Population structure analysis by PCA and MDS showed *P. falciparum* from all seven geo-temporal populations clustering into a single genetic cluster with a few outliers (Figures 2A,B). The first two components of PCA, respectively, accounted for 34 and 31% of the total observed variation among infections. No significant population substructure was evident by displaying other components from PCA and MDS (Supplementary Figure S4). Only a small number of outliers, mostly from the 2015/2016 collections from Nioro, were observed beyond the main PCA cluster. However, cross-validation for the number of ancestral groupings plateaued at *k* = 3, suggesting three ancestral populations. Admixture analysis with *tess3r* showed all samples as an admixture of the three ancestries except for those collected from Kolle in 2007, which predominantly had two ancestries (Figure 2C).

### Drug resistance and antigen loci differentiate geo-temporal and temporal populations

**FIGURE 3:**
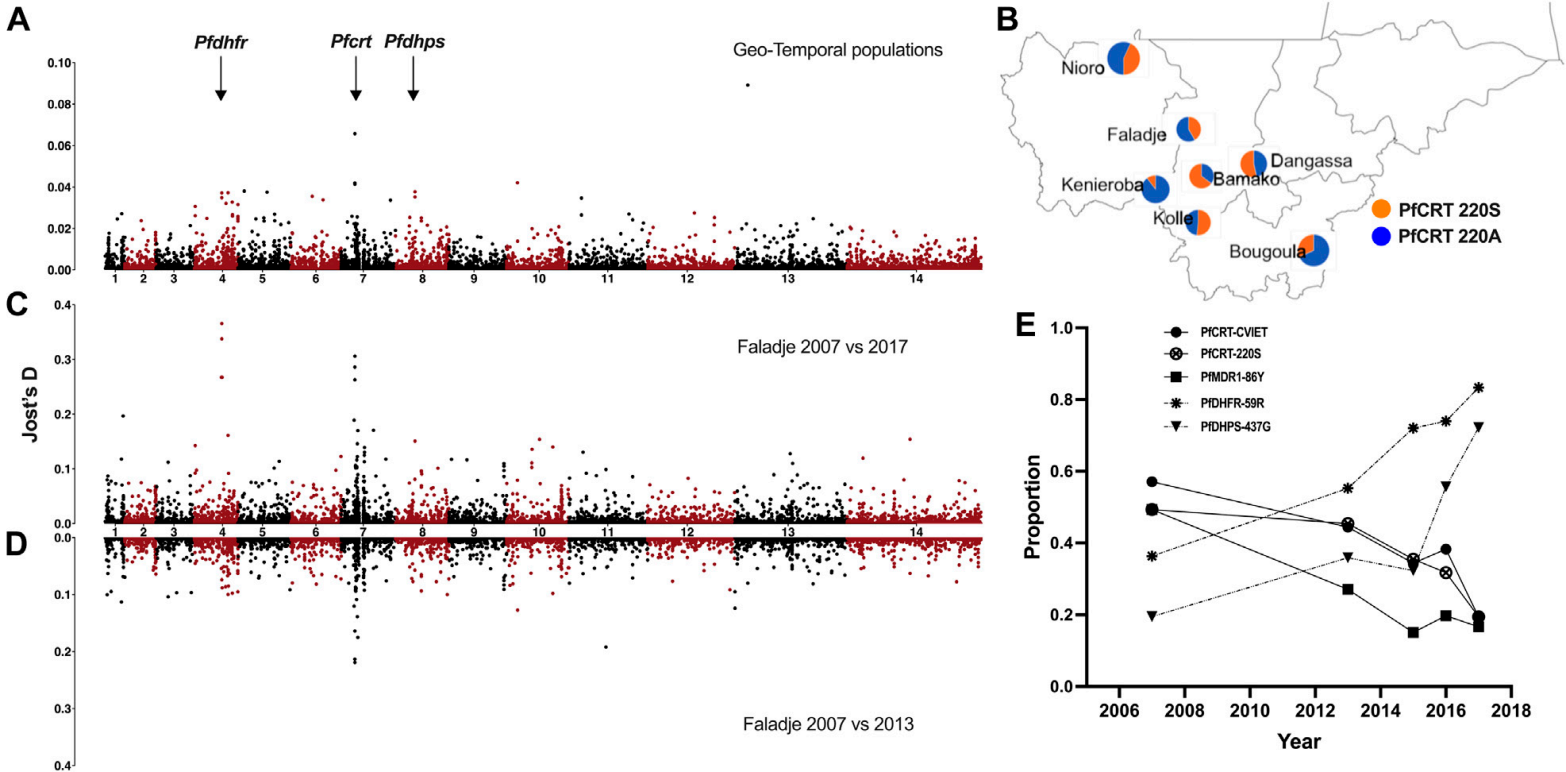
Genome-wide SNP differentiation between geo-temporal populations of *P. falciparum* from Mali. **(A)** Manhattan plot of genome-wide Jost’s D differentiation index per SNP between all sampling locations (geo-temporal populations) in Mali. The genomic location of drug-resistance-associated genes (*pfdhfr*, *pfcrt*, and *pfdhps*) is indicated with arrows. **(B)** Allele frequency proportions (pie charts) for the PFCRT A220S locus across different sampling locations. **(C)** Manhattan plot of genome-wide Jost’s D differentiation index per SNP between samples from Faladje collected in 2007 and 2015–2017 and **(D)** Faladje 2007 against 2013. **(E)** Temporal changes in proportion of alleles and haplotypes of drug-resistance-associated loci in *pfdhfr*, *pfcrt*, and *pfdhps*.

As the lack of structure between populations could be due to geneflow, we assessed *P. falciparum* geneflow or differentiation between pairs of sampling sites (geo-temporal populations) and between all temporal samples or from Faladje only; 2007 against 2013 and 2007 against combined samples from 2015, 2016, and 2017 using differentiation indices (F_ST_ and Jost’s D). Genome-wide pairwise F_ST_ and Jost’s D were generally low between geo-temporal and temporal population pairs (Supplementary Table S3). Isolates from Bamako and Faladje, mostly collected from 2013, were the least differentiated. Except against Faladje and Kolle (samples in 2007), pairwise differences were highest when Bamako isolates (mostly from 2013) were compared to those from Bougoula-Hameau, Kéniéroba, Nioro, and Dangassa. Sampling site differences were, therefore, confounded by temporal heterogeneity. These geo-temporal populations were most differentiated at SNP located at position 404407 of chromosome 7 (Pf3D7_07_v3: 404407), which codes for codon A220S of the chloroquine-resistant transporter (*pfcrt*)*,* and Pf3D7_13_v3.400162 that codes for L264V in PF3D7_1308600 conserved protein of unknown function on chromosome 13 (Figure 3A). The prevalence of the major PFCRT 220S variant was significantly higher (*p*-value = 3.197e-15) in 2013 samples from Bamako (65% of infections), while the alternative 220A variant was most frequent in 2016 samples from Kenieroba (Figure 3B). However, the strongest differentiating SNPs were observed between 2007 against 2015–2017 temporal samples from Faladje, led by Pf3D7_04_v3.748262 and Pf3D7_04_v3.748239 which code for SP resistance-associated PFDHFR codons, R59C and I51N, respectively (Figure 3C). These were followed by Pf3D7_07_v3.404836 and Pf3D7_07_v3.403625, which code for PFCRT E271Q and K79T, respectively, and differentiated 2007 and 2013 samples (Figure 3D). In Faladje, the PFCRT 220S variant declined in prevalence, paralleling the decline of PFCRT quinoline resistance haplotype (CVIET) at codons 72–76 (Figure 3E). SNPs in the other known quinoline-resistance-associated gene, *pfmdr1*, were not outliers by differentiation indices. However, the PFMDR1 86Y variant also declined in prevalence from 40% in samples from 2007 to less than 20% in the most recent 2017 samples. The decline in quinoline markers contrasted the increase in isolates with SP resistance variants, PFDHFR 59R and PFDHPS 437G across the decade of sampling.

Considering the top 0.5% of per locus Jost’s D distribution between each geo-temporal or temporal population pair, 185 SNPs (126 coding for non-synonymous variants) were identified as relatively stronger drivers of differences in allele frequencies (Supplementary Table S4). These were distributed in 137 genes involved in several biological roles (Supplementary Figure S5). Twenty-five of these genes are annotated as invasion ligands or antigens and four of them were genes associated with drug resistance (*pfcrt*, *pfpare*, *pfdhfr*, and *pfdhps*)*.* The antigenic loci differentiating geo-temporal populations were mostly those coding for members of dominant gene families in blood stages, including erythrocyte/Duffy binding proteins (EBA/DBL), merozoite surface proteins (MSP), cytoadherence proteins (CLAG), surface-associated proteins (SURFINS), hypervariable exported proteins (HYPs), early-transcribed membrane protein (ETRAMP), a reticulocyte-binding protein (*pfrh1*), and protein E140 (PF3D7_0104100). The highest number of differentiating SNPs was in *pfhyp15* (five non-synonymous SNPs). Differentiating SNPs were also identified in gametocyte-associated genes, *pfgig* and *pfgexp05*, as well as *pfcap93* which play a role in sporozoite development.

### Allele frequency correlation and linkage disequilibrium between SNPs at drug resistance markers

Thirty-two SNPs in selected drug resistance genes (*pfcrt*, *pfdhps*, *pfdhfr*, *pfmdr1, pfmdr2,* and *pfk13*) attained a frequency of 2% or above in at least one of the geo-temporal populations (Table 1). Cumulatively, infections from 2013 collected in the urban site, Bamako, had the highest proportion of mutations in drug-resistance-associated genes. However, the allele frequency patterns between sites strongly correlated positively, exceeding an *r* ^2^ of 0.9 between most pairs except against Kolle, where samples were from 2007 (Supplementary Figure S6). Though the frequency patterns of these resistance-associated mutations correlated between geo-temporal populations, significant differences in the proportion of infections with mutant alleles were observed for PFCRT K76T (*p* = 0.003919), A220S (*p* = 0.000009416), *Q*271E (*p* = 0.001798), I356T (*p* = 0.0002915), R371I (*p* < 2.2*10^−16^), and PFMDR1 N86Y (*p* = 0.01123). For antifolate resistance markers, high frequencies (mean 70%) of PFDHFR mutants 51I, 59R, and 108N were observed across all sampling sites. None of the common artemisinin-resistance-associated variants in *pfk13* were detected, though four nonsynonymous SNPs coding for PFK13 R255K, K189N, K189T, and G112E in the non-propeller domain of the Kelch 13 protein were detected. PFK13 K189T was detected in over 50% of isolates from all geo-temporal populations.

**TABLE 1:** Percentage of isolates in Mali with mutant variants within known *Plasmodium falciparum* drug-resistance-associated genes. Only variants found in at least 2 percent of any of the geo-temporal populations are presented.

Gene	Mutation	Position	Bamako	Bougoula	Dangassa	Faladje	Kenieroba	Kolle	Nioro
CRT	K76T	Pf3D7_07_v3.403625	64	27	50	37	48	57	46
	A220S	Pf3D7_07_v3.404407	66	29	53	38	23	59	49
	Q271E	Pf3D7_07_v3.404836	66	28	53	38	50	62	48
	N326S	Pf3D7_07_v3.405362	2	3	1	1	2	0	0
	I356T	Pf3D7_07_v3.405600	39	12	39	22	39	24	41
	R371I	Pf3D7_07_v3.405838	65	0	0	48	0	61	0
DHFR	N51I	Pf3D7_04_v3.748239	63	77	76	72	76	51	70
	C59R	Pf3D7_04_v3.748262	68	83	79	73	80	57	72
	S108N	Pf3D7_04_v3.748410	69	85	83	72	81	57	72
DHPS	E189Q	Pf3D7_08_v3.548940	1	1	2	1	1	3	1
	I431V	Pf3D7_08_v3.549666	1	2	0	0	0	0	1
	S436A	Pf3D7_08_v3.549681	57	58	46	53	42	65	56
	G437A	Pf3D7_08_v3.549685	47	35	30	48	37	63	55
	A613S	Pf3D7_08_v3.550212	7	8	9	6	7	10	3
MDR1	N86Y	Pf3D7_05_v3.958145	22	11	11	14	17	29	12
	Y184F	Pf3D7_05_v3.958440	66	58	63	62	68	71	60
	P203S	Pf3D7_05_v3.958496	1	2	1	0	0	1	1
	N504K	Pf3D7_05_v3.959401	4	0	0	4	0	1	0
	F938Y	Pf3D7_05_v3.960702	4	7	3	2	1	0	0
	D1246Y	Pf3D7_05_v3.961625	1	3	1	2	1	2	1
MDR2	M1019K	Pf3D7_14_v3.1954620	0	0	0	3	0	0	0
	I492V	Pf3D7_14_v3.1956202	52	62	51	53	51	60	44
	F423Y	Pf3D7_14_v3.1956408	76	75	64	66	63	75	57
	K253T	Pf3D7_14_v3.1956918	2	1	4	4	1	1	6
	V250I	Pf3D7_14_v3.1956928	3	9	4	3	5	3	3
	S208N	Pf3D7_14_v3.1957053	89	83	87	83	87	96	85
	R176L	Pf3D7_14_v3.1957149	8	0	0	9	0	9	0
	T23M	Pf3D7_14_v3.1957608	1	2	2	4	1	1	3
K13	R255K	Pf3D7_13_v3.1726234	2	0	1	2	2	5	5
	K189N	Pf3D7_13_v3.1726431	5	2	5	2	6	9	2
	K189T	Pf3D7_13_v3.1726432	51	50	53	54	54	52	43
	G112E	Pf3D7_13_v3.1726663	2	0	1	0	1	0	4

**FIGURE 4:**
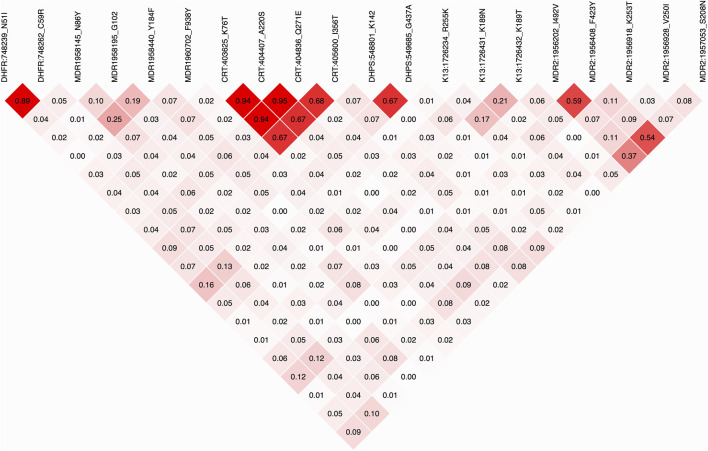
Heatmap of linkage disequilibrium (LD) between SNPs in drug resistance markers, labeled by gene name, genomic position, and amino acid allele. Each cell is color shaded by intensity of LD, with the *r* ^2^ value between the loci indicated.

For alleles in pairs of drug resistance genes, the strongest positive correlation of overall frequencies was between alternative alleles in SNPs coding for PFMDR1 Y184F, PFK13 K189N, PFMDR1 N86Y PFMDR2 S208N, and PFDHPS E189Q. The frequency of these alleles also positively correlated with PFDHPS G437A, PFK13 R255K, PFCRT A220S, PFCRT K76T, and PFCRT Q271E. Both sets of loci negatively correlated with PFDHFR N51I, PFDHFR C59R, PFDHFR S108N, PFMDR2 V250I, PFCRT N236S, and PFMDR1 F938Y (Supplementary Figure S7). Surprisingly, the strongest negative correlation of allele frequencies was between mutations in quinoline resistance markers PFMDR1 D1246Y and PFCRT I356T. Likewise, the frequency of PFDHFR S108N negatively correlated with PFDHPS G437S, both of which are markers of antifolate resistance. The correlation in allele frequencies was evident as linkage disequilibrium (LD) in variants within and between genes, even for those located on different chromosomes (Figure 4). The strongest LD was for SNPs in *pfcrt*, *pfdhfr*, *pfdhps*, and *pfmdr2*. The weakest within-gene LD was observed for SNPs in *pfmdr1* and *pfk13*. Cross-genic LD was weaker but evident between *pfdhfr* and *pfdhps* SNPs, both of which are markers of antifolate resistance. Two *pfdhfr* SNPs coding for N51I and C59R were in relatively stronger LD with *pfmdr2* SNPs coding for F423Y and S208N. *Pfmdr1* Y184F also showed relatively higher LD with the *pfk13* variant coding for K189N, though the significance compared to the background was not determined. Relatively higher LD was also detected between *Pfmdr2* variants and those in all other drug resistance markers.

### Positive selection signatures at drug resistance and antigenic loci

**FIGURE 5:**
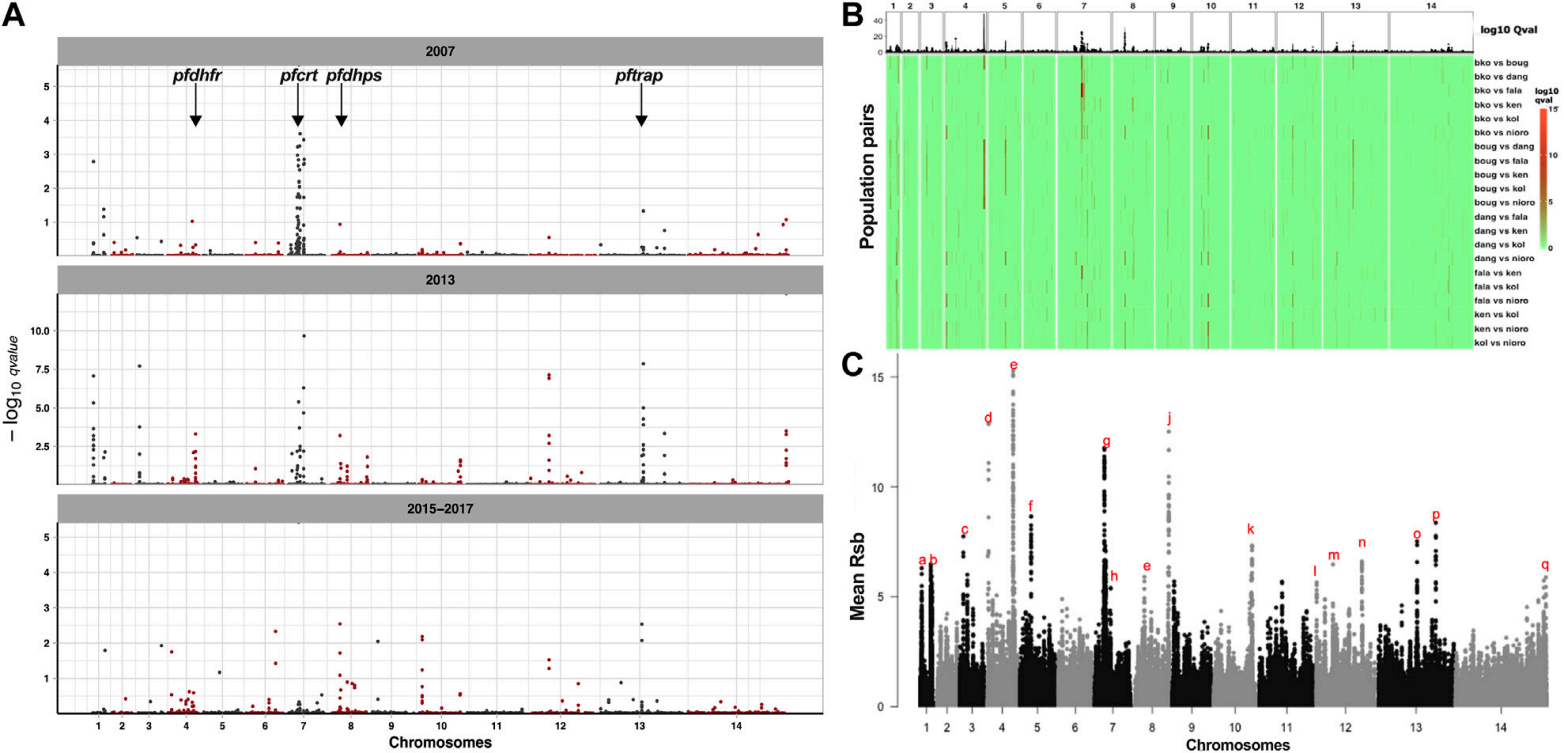
Signatures of positive selection in the genomes of temporal and geo-temporal populations of *P. falciparum* in Mali. **(A)** Manhattan plots of genome-wide −log10 q-values of standardized integrated haplotype score (iHS) for temporal populations from Faladje, showing positions of genes for drug-resistance-associated loci, *pfdhfr*, *pfcrt*, and *pfdhps,* and the antigen *pftrap.* Each row represents the year of sampling, showing 2007, 2013, and combined 2015–2017 samples. **(B)** Heatmap of -log10 q-values of cross population extended haplotype homozygosity test between pairs of geo-temporal populations shown in rows with abbreviated names for Kolle (kol), Bougoula-Hameau (boug), Faladje (fala), Kenieroba (ken), Bamako (bko), Dangassa (dang), and Nioro (nioro). **(C)** Combined Rsb scores of SNPs across the *P. falciparum* genome over all pairwise comparison of seven geo-temporal populations. The top genomic regions harboring SNPs with a mean Rsb >5 are labeled alphabetically from chromosome 1–14. The genomic coordinates and genic loci within these regions are given in Table 2.

High LD between variants in drug resistance markers is suggestive of a high frequency of a small number of haplotypes that could be because of positive selection. Based on a threshold value of at least 1 for the -log10 q-value of standardized integrated haplotype score (|iHS|), 143 out of 9,359 common SNPs in 19 genomic regions and 53 genes, marked candidate signatures of selective sweeps across the seven geo-temporal populations (Supplementary Figure S8). Considering individual geo-temporal populations, 186 SNPs in 56 genes marked regions of candidate selective sweeps in at least one population (Supplementary Table S5). Together, the identified genes were dominated by those involved in cell cycle, response to a stimulus or antigenic surface loci involved in cell movement (invasion, gliding), (Supplementary Figure S9). The highest density of sweep variants was found on chromosome 7 around the *pfcrt* locus, though no *pfcrt* SNP met the 99.5 percentile threshold for outliers. The SNPs in the *pfcrt* sweep region were mostly downstream and included 13 SNPs coding for a putative mediator of RNA polymerase II transcription subunit 14 (PF3D7_0709300). Selective sweeps were also evident around *pfdhfr* and *pfdhps*, markers of SP resistance. Several antigens were also identified with sweep signatures, with the highest number of sweep SNPs within the *pftrap* (PF3D7_1335900) coding region. Considering only the temporal populations from Faladje, regions of selective sweeps were similar to those described for geo-temporal populations (Figure 5A). These regions were found on chromosome 1 around the immune modulating protein E140 (PF3D7_0104100), *pfdhfr* on chromosome 4, *pfcrt* gene on chromosome 7, cell traversal protein for ookinetes and sporozoites (CELTOS) on chromosome 12, thrombospondin-related anonymous protein (TRAP) on chromosome 13, and a conserved *Plasmodium* protein (PF3D7_1475800) on chromosome 14, (Supplementary Table S6). Together, these regions of selective sweeps detected by iHS code for proteins predominantly involved in nucleic acid metabolism, chromosome structure and segregation, entry into the host cell, and immune responses.

### Differences in signatures of positive selection between pairs of populations

Differences in haplotypes and regions of selective sweeps between geo-temporal populations can be distinguished from comparing extended haplotype homozygosity between populations, captured by the cross-population metrics Rsb and cross XP-EHH. Both Rsb and XP-EHH metrics identified several regions of selective sweeps as differentiating geo-temporal populations (Figure 5B, Supplementary Figure S10). Overall, 32 regions on 13 chromosomes, except for chromosome 2, showed differences in the range of extended haplotypes between pairs of populations (Figure 5C; Table 2, Supplementary Tables S7, S8). The strongest signals were in regions coding for antigenic families (*pfphist*, *pfhyp*, *pfsurfins*, and *pfmsp*), candidate vaccine targets (*pfcsp*, *pfceltos*, *pflsa1*, and *pftrap*), and housekeeping genes as well as known drug resistance loci. For the latter, the widest span was the region on chromosome 7, around the chloroquine resistance transporter (*pfcrt*). The discriminating genomic loci around *pfcrt* were mostly observed when the largest number of isolates collected from Bamako in 2013 was compared to those from other sites. Additional positive selection regions differentiating populations were within the vicinity of other known drug resistance genes, *pfdhfr* on chromosome 4, *pfmdr1* on chromosome 5, and *pfdhps* on chromosome 8. These latter differences were relatively stronger when isolates from Bougoula were compared to other geo-temporal populations.

**TABLE 2:** *Plasmodium falciparum* genomic regions with differentiating signatures of positive selection from Figure 5C. Only segments with clusters of SNPs in selective sweep regions and between population Rsb -log10 *p*-value of at least 5, and the genes coded for are presented.

Rsb region	Genomic location	Gene ID (number of genes)	Known gene symbol
a	Pf3D7_01_v3:180285-180293	PF3D7_0104100 [1]	Protein E140, putative
b	Pf3D7_01_v3:495680-528907	PF3D7_0113100 - PF3D7_0113800 [8]	SURFIN1.1, SURFIN1.2, HYP1, HSP40-II, DBL-containing protein
c	Pf3D7_03_v3:221567-221577	PF3D7_0308100 [1]	CSP
d	Pf3D7_04_v3:110070-140834	PF3D7_0401900 - PF3D7_0402300 [4]	ACS6, Phista, Phistb, SURFIN4.1, RH1
e	Pf3D7_04_v3:989334-1035251	PF3D7_0421700 - PF3D7_0422500 [9]	emp24, EMAA, a-tub2, 40S RP S19, BRR2
f	Pf3D7_05_v3:476551-477676	PF3D7_0511300 [1]	MORN repeat protein
g	Pf3D7_07_v3:375770-465691	PF3D7_0708200 - PF3D7_0710200 [23]	HSP90, HSP110, HSP86, SCO1, CRT, CG1, CG7, MED14
			PARE
h	Pf3D7_07_v3:635335-635689	PF3D7_0713900 [1]	Conserved *Plasmodium* protein
I	Pf3D7_08_v3:468950-469018	PF3D7_0809200 [1]	Pfa55-14
J	Pf3D7_08_v3:1290857-1315811	PF3D7_0830300 - PF3D7_0830800 [6]	SIAP2, SURFIN 8.2, PHISTc, HYP9, SLS-TRP
k	Pf3D7_10_v3:1386709-1447856	PF3D7_1035000 - PF3D7_1036700 [16]	GLURP, MSP3, MSP6, MSP, MSP11 DBLMSP, DBLMSP2, LSA1
l	Pf3D7_12_v3:96273-96385	PF3D7_1201400 [1]	*Plasmodium* exported protein
m	Pf3D7_12_v3:659919-659920	PF3D7_1216600 [1]	CelTOS
n	Pf3D7_12_v3:1662107-1663492	PF3D7_1239800 [1]	Conserved *Plasmodium* protein
o	Pf3D7_13_v3:1465494-1466264	PF3D7_1335900 [1]	TRAP
p	Pf3D7_13_v3:2114559-2114619	PF3D7_1352900 [1]	*Plasmodium* exported protein
q	Pf3D7_14_v3:3121408-3193573	PF3D7_1475800 - PF3D7_1477600 [19]	KELT, ETRAMP14.2, PHIST, HYP, FIKK, SURFIN 14.1

### Common signatures of balancing selection dominated by antigenic markers

Genetic variation patterns in antigenic loci can be indicative of balancing selection, identifiable with allele-frequency-based summary statistics such as Tajima’s D for each gene. The overall distribution of gene-wise Tajima’s D was skewed to negative values for most genes across all geo-temporal populations (Supplementary Figure S11). The Tajima’s D values between all pairs of populations were strongly correlated (Pearson’s coefficient >0.8), identifying similar loci as common targets of balancing selection (Supplementary Figures S12, Supplementary Table S9). There were 15 genes that showed highly positive Tajima’s D (>2.5) in at least two populations (Table 3). Of these, Pf3D7_0221000 (*Plasmodium* exported protein) and PF3D7_1004800 (ADP, ATP carrier protein) were under balancing selection in most populations, while Duffy binding-like merozoite surface protein 2 (DBLMSP2, PF3D7_1036300) had the highest Tajima’s D value but not for all populations. Amongst the top 15 common genes were the invasion ligands, *pfdblmsp2* and *pfsurfin8.2*, as well as the drug resistance gene, *pfcrt,* and other loci involved in metabolic processes and environmental responses.

**TABLE 3:** Genes with consistent signatures of balancing selection across geo-temporal Malian *P. falciparum* populations, based on a Tajima’s D value of at least 3 in one or more populations. The number of geo-temporal population pairs (popPairs) and mean Tajima’s D value for the gene across the populations are presented.

Gene	Protein description	PopPairs	Mean TjD
PF3D7_0221000	*Plasmodium* exported protein, unknown function	21	3.99
PF3D7_0420200	Holo-[acyl-carrier-protein] synthase, putative (ACPS)	15	3.17
PF3D7_0422800	Serpentine receptor 12, putative (SR12)	10	3.42
PF3D7_0619600	Conserved *Plasmodium* protein, unknown function	1	3.07
PF3D7_0630600	Deubiquitinating enzyme MINDY, putative	10	3.20
PF3D7_0709000	Chloroquine resistance transporter (CRT)	1	2.74
PF3D7_0710200	Conserved *Plasmodium* protein, unknown function	3	3.30
PF3D7_0728900	RNA-binding protein, putative	1	3.14
PF3D7_0729700	Zinc finger protein, putative	1	2.95
PF3D7_0830800	Surface-associated interspersed protein 8.2 (SURFIN 8.2)	10	3.19
PF3D7_1004800	ADP, ATP carrier protein 2 (AAC2)	21	3.03
PF3D7_1036300	Duffy binding-like merozoite surface protein 2 (DBLMSP2)	6	4.41
PF3D7_1302900	Conserved protein, unknown function	15	3.40
PF3D7_1402900	Conserved *Plasmodium* protein, unknown function	3	2.86
PF3D7_1409700	Conserved protein, unknown function	1	2.97

## Discussion

Malaria transmission intensity in Mali is one of the highest in West Africa and dominated by the *P. falciparum* parasite. Though the country is divided by the River Niger, and there is heterogeneity in transmission levels, the analysis here showed that the malaria parasite populations across seven endemic sites share the same genetic structure. This provides evidence of temporal and geographic geneflow across the southern transmission belt of the country, similar to what has been described for the parasite from other malarial endemic settings in West Africa (Mobegi et al., 2012). As the sampling period spanned a decade of interventions, the complexity of infection determined by the inbreeding index (*F*_WS_) varied between populations partly driven by transmission differences. Infections from the region of Kolle were the most complex (polygenomic), while those from Nioro and the metropolis of Bamako were predominantly clonal. The samples from Kolle were the oldest in the collection and the higher complexity likely reflected the higher transmission rates in 2007. The complexity of infection can track changes in transmission imposed by intervention, and this was evident when isolates from Faladje showed a slight but significant reduction in complexity between 2007 and 2017. The association between the complexity of infection and transmission intensity could also be driven by the difference in *Anopheles* vector densities between rural and urban dwellings (Bendixen et al., 2001). Previous studies have reported lower infection rates and vector densities in Bamako and Nioro compared to the other sites included in this study (Sissoko et al., 2017; Cissoko et al., 2022). Thus, Nioro had the least complex infections but also the shortest transmission season because it is bordering the Sahara. On the other hand, Bamako is urban, and most infections could be resulting from a limited number of sources, some of which could be imported from higher transmission regions by migration. Future analysis of genetic relatedness and transmission networks could show the flow of infections between these sites.

Differences in transmission could affect the health-seeking behavior and use of drugs, especially with malaria, where presumptive treatment and self-medication remain common (Thera et al., 2000; Graz et al., 2015). Despite evidence of a single genetic cluster and overall low differentiation indices (*F*_ST_ and Jost’s D), the strongest differences in allele frequencies were for SNPs within or around drug-resistance-associated and immunity-related markers. This includes codon position A220S in the *pfcrt*, a gene involved in parasite resistance to chloroquine (Fidock et al., 2000). Codon 220 of *pfcrt* is located within a transmembrane region and the 220S variant is found in chloroquine-resistant *P. falciparum* strains such as Dd2 and FCB (Gabryszewski et al., 2016). It is strongly linked to the 76T mutant and the CVIET haplotype that is most associated with chloroquine resistance. The highest frequency of this variant was found in Bamako isolates collected in 2013, where they could have been under selection from continuous chloroquine use or from other quinolines such as amodiaquine that are used in seasonal malaria chemoprevention (Djimde et al., 2010; Thera et al., 2018). As sites furthest from Bamako with more recent samples had higher frequencies of the reference/wildtype variant, reversal to wildtype variants driven by discontinuation of chloroquine seem the most likely explanation. The first-line treatment for malaria in Mali, similar to most of West Africa is artemether–lumefantrine, an artemisinin combination therapy (ACT). These have been used for over a decade but surprisingly have not selected for the wild variant in *pfcrt* in some regions such as Bamako as reported for other parts of Africa (Ehrlich et al., 2021)*.* However, lumefantrine selects for wildtype variants at *pfcrt* and *pfmdr1* and this was evident with the reduction in the mutant allele frequencies when isolates from Faladje were analyzed across the decade. In fact, the wildtype (N allele) variant at *Pfmdr1* codon 86 but the mutant (F allele) at codon 184 was at higher frequencies across all sites, probably selected for by lumefantrine (Maiga et al., 2021). The combination of these alleles has been shown to significantly affect treatment responses and post-treatment protection by ACTs (Venkatesan et al., 2014; Bretscher et al., 2020). Although none of the potent artemisinin resistance variants were detected, mutations at PFK13 R255K, and K189T were detected, the latter at frequencies above 50% in most sites. These mutations have been found in Asian isolates, and though not shown to affect the ring survival phenotype associated with delayed clearance following artemisinin treatment, their higher linkage disequilibrium with *pfmdr1* and *pfcrt* variants suggest that they may form the background for any future selection or emergence of ACT resistance variants. In addition to ACTs, Sahel countries such as Mali use sulfadoxine–pyrimethamine and amodiaquine for chemoprevention in children and SP in pregnant women (Thera et al., 2018; Sangho et al., 2021). These drugs also continue to select for mutant variants, with a temporal increase in the frequencies of antifolate-resistance-associated mutations in *pfdhfr* and *pfdhps* as shown in neighboring Senegal (Ndiaye et al., 2013). Monitoring their dynamics and how they combine with other variants associated with resistance to ACT partner drugs need to be considered for micro-stratification, customization, and optimization of malaria drug interventions and elimination strategies in Mali.

Selection from drugs and other interventions leaves signatures on the genome that can distinguish their impact on populations. From extended haplotype homozygosity analysis (|iHS|), several genomic regions showed evidence of recent directional selection in Mali. This included strong signatures around the antimalarial drug resistance gene *pfcrt* on chromosome 7, and antifolate resistance markers *pfdhfr* and *pfdhps* on chromosomes 4 and 8, respectively. The signals detected by these analyses are indicative of the extensive historic use of chloroquine and sulfadoxine–pyrimethamine as antimalarial therapies in Mali before the introduction of ACTs. Similarly, studies conducted across Africa have shown the commonality of strong signatures of selection related to the use of chloroquine and sulfadoxine–pyrimethamine (Mobegi et al., 2012; Amambua-Ngwa et al., 2019). Likewise, strong signatures were also detected around vaccine candidate genes including the thrombospondin-related anonymous protein (TRAP) on chromosome 13, a protein involved in sporozoite binding to hepatic cells and the gene encoding the cell transversal protein for ookinete and sporozoites (CELTOS, PF3D7_1475800) on chromosome 14 (Amambua-Ngwa et al., 2018). Not all populations showed signs of selection to the same extent, as there were differences in type and extent of haplotypes as determined by cross-population-based tests, Rsb and XP-EHH. Indeed, several known antigens, such as protein E140 (PF3D7_0104100) and surface and invasion ligands had SNPs differentiating geo-temporal populations, despite the absence of selective sweep signatures. Protein E140 is a novel malaria antigen that has been shown in the mouse parasite *Plasmodium yoelii* to induce antibody-mediated sterile immunity and, like other antigens, it is being considered for inclusion in future multivalent candidate vaccines (Smith et al., 2020). Other differentiating genes, such as *pfgig* and *pfgexp02*, participate in gametocytogenesis, a process also targeted for vaccine development (Gardiner et al., 2005). Hence, the forces that are driving the rise or decay of these signatures could be heterogeneous, or the levels of selection could be affected by differences in recombination from variance in transmission intensity, immunity, and population diversity as interventions were implemented over the decade. Indeed, SNPs in antigenic loci showed relatively stronger differentiation between temporal and geo-temporal samples, an indication of the effect of variation in immune selection between populations. This is important in view of the future application of malaria vaccines in Africa. Already, the WHO-approved RTS,S vaccine, which contains the repeat and C-terminal regions of the *P. falciparum* circumsporozoite protein (CSP) selects for non-vaccine variants in the population and the observed genetic variation in this study could further affect its efficacy in Malian populations (Neafsey et al., 2015).

Immune action also mounts selection pressure on parasite populations, which leaves signatures of balancing selection in the genomes. It was not surprising to find the *Pfdblmsp2* gene harboring the strongest signature of balancing selection by most populations. It has been identified previously as a target of balancing selection despite a potential role in drug responses (Van Tyne et al., 2011; Amambua-Ngwa et al., 2012). Balancing selection signatures were also observed in several known antigen classes. These results are important for the further development of these proteins as components of future multi-antigenic vaccines. Members of immune and invasion proteins such as CLAG and SURFIN families showed signatures indicative of balancing and directional selection acting at the same time. This was also observed for the *pfcrt* drug resistance gene, suggesting that dual selection from drugs and probably immunity may be acting on these loci as previously shown (Samad et al., 2015). It is, therefore, important to consider the variance and extent of haplotypes across malarial populations as candidate loci with dual selection are considered for inclusion in future multivalent vaccines. As one of the limitations of this study is the temporal heterogeneity of the sampling, a prospective survey to include recent isolates from more sites and the northeast would be informative and help in spatially planning genomic surveillance approaches for malaria elimination in Mali.

## Conclusion

Infections of *Plasmodium falciparum* across Mali are genetically similar but complex within a decade of sampling. The genetic difference between temporal or geographic populations targeted drug resistance and immune genes affected by drug interventions and differences in immunity imposed by variation in transmission intensities. As drugs and future vaccines are vital tools for elimination of malaria, the temporal and spatial population-specific differences in associated alleles should be monitored for exploitation as current and new malaria elimination strategies are being implemented.

## Data Availability

The datasets presented in this study can be found in European Nucleotide Archives (ENA). The names and sample accession number(s) can be found in the article/Supplementary Material.
