# Supplementary material for: Genome-wide SNP analysis of Plasmodium falciparum shows differentiation at drug-resistance-associated loci among malaria transmission settings in southern Mali
Source: Front Genet. 2022 Oct 4;13:943445. doi: 10.3389/fgene.2022.943445 (PMC9576839; doi:10.3389/fgene.2022.943445)
Supplement: Supplementary file 1 [file DataSheet1.PDF]

Supplementary information

Genome-wide SNP analysis of *Plasmodium falciparum* shows differentiation at drug resistance associated loci among malaria transmission settings in Southern Mali

Aoua Coulibaly[1,3], Mouhamadou Fadel Diop[5], Aminatou Kone[1], Antoine Dara[1], Amed Ouattara[1,2], Nicola Mulder[3], Olivo Miotto[4], Mahamadou Diakite[1], Abdoulaye Djimde[1], Alfred Amambua-Ngwa[5*]

[1]*University of Science, Techniques, and Technologies of Bamako, Bamako, Mali*
[2]*University of Maryland Baltimore, Baltimore, MD*
[3]*University of Cape Town, Cape Town, South Africa*
[4]*Mahidol University, Bangkok, Thailand*
[5]*Medical Research Council Unit The Gambia at LSHTM, Banjul, The Gambia*

*Corresponding author. Email: alfred.ngwa@lshtm.ac.uk; angwa@mrc.gm

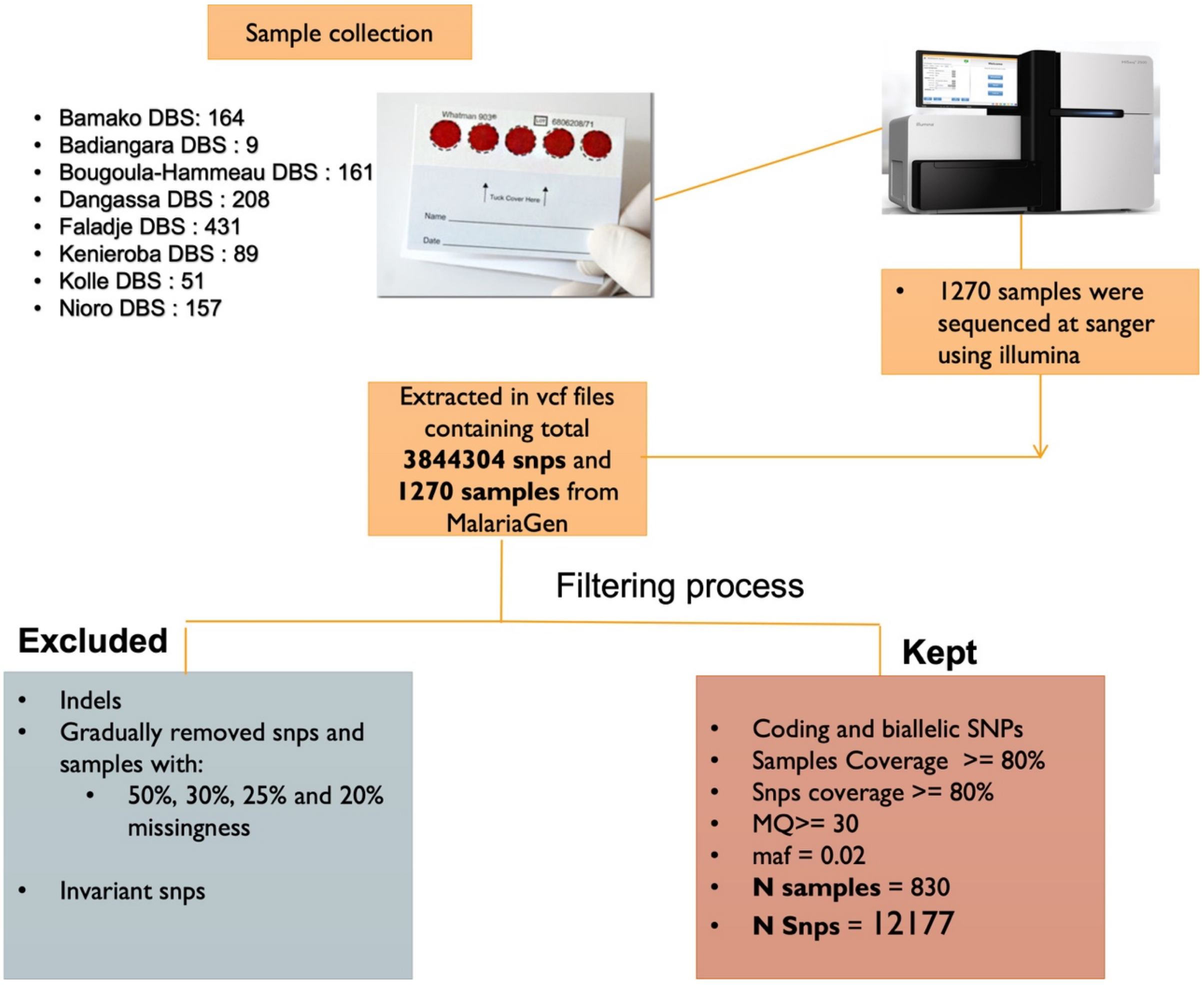

Figure S1. Samples, sequencing, and data filtration. Illumina derived genomic variants in a vcf file were filtered using vcftools commands to retain 12,177 bi-allelic SNPs from 830 *P. falciparum* isolates.

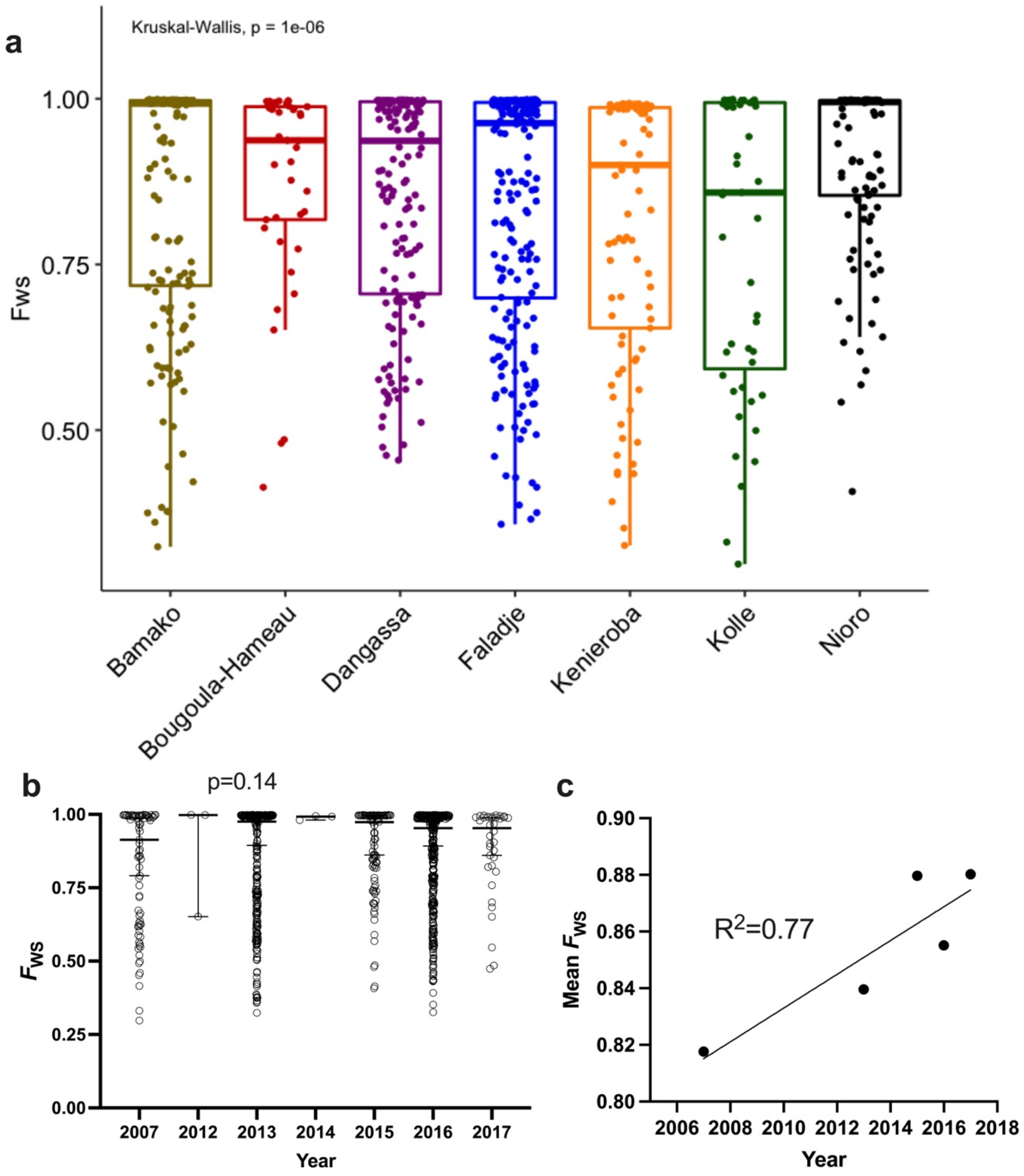

Figure S2. Distribution of complexity of *Plasmodium falciparum* infections in different regions, shown as box plots (a) and distribution per year of sampling (b) in Mali. The distribution of F_{WS} scores was significantly different across isolates from different geographic populations (Kruskal-Wallis, p = 1e-06) but not from temporal populations (p=0.14). There was a significant trend (p=0.043) towards increasing F_{WS} in more recent samples (c).

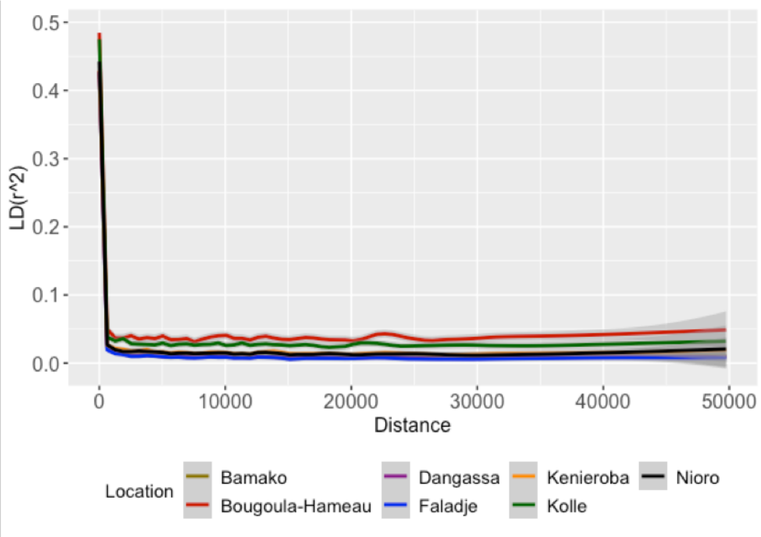

Figure S3. Decay of linkage disequilibrium in seven geo-temporal populations in Mali. Linkage disequilibrium between 12,171 coding SNPs was low in all seven populations, decaying rapidly below an r^2 of 0.05 within 10000 base pairs. We observe a linkage at 23000 base pairs in both Bougoula and Kolle.

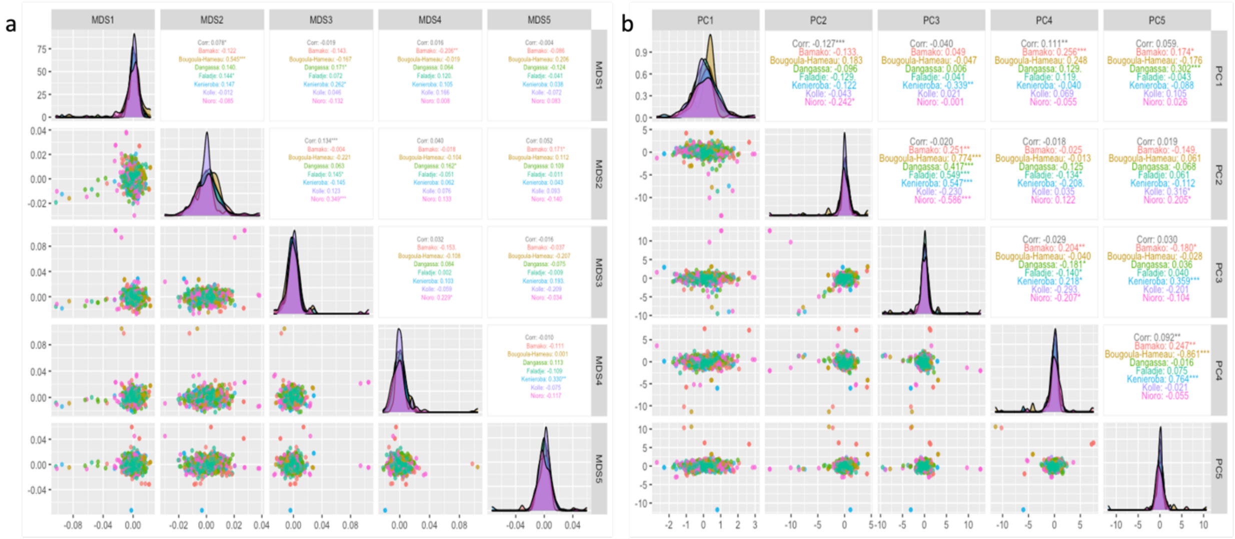

Figure S4. Pairwise scatter matrices of a) Multidimensional scaling (MDS) axes 1 to 5 and, b) Principal components analysis (PCA) axes 1 to 5. Each box in the lower triangle shows points per isolate for pairs of axes. The upper triangle shows the correlation between coordinates plotted, and the diagonal is the distribution of the coordinates plotted.

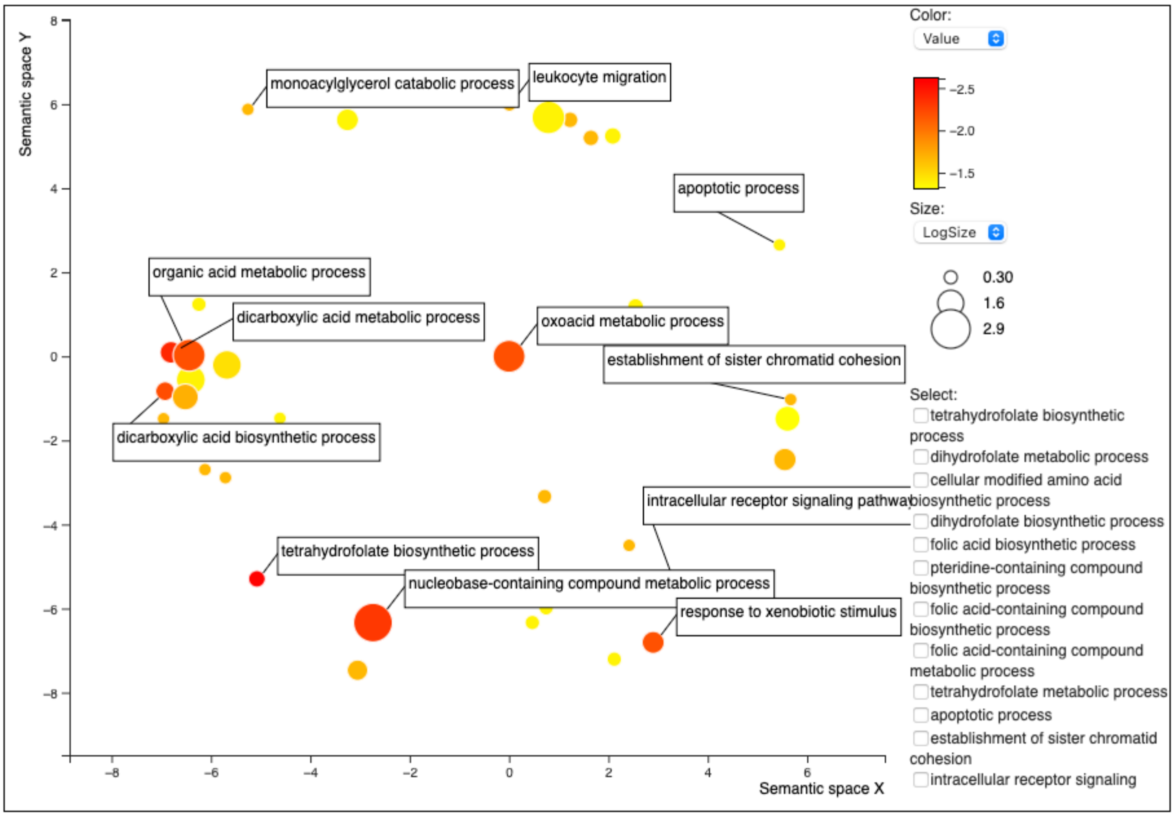

Figure S5. Semantic clusters for Gene ontologies of genes differentiating temporal populations from Faladje. Each point is shaded from yellow to red depending on the significance with the size of the points being proportionate to number of genes supporting the function.

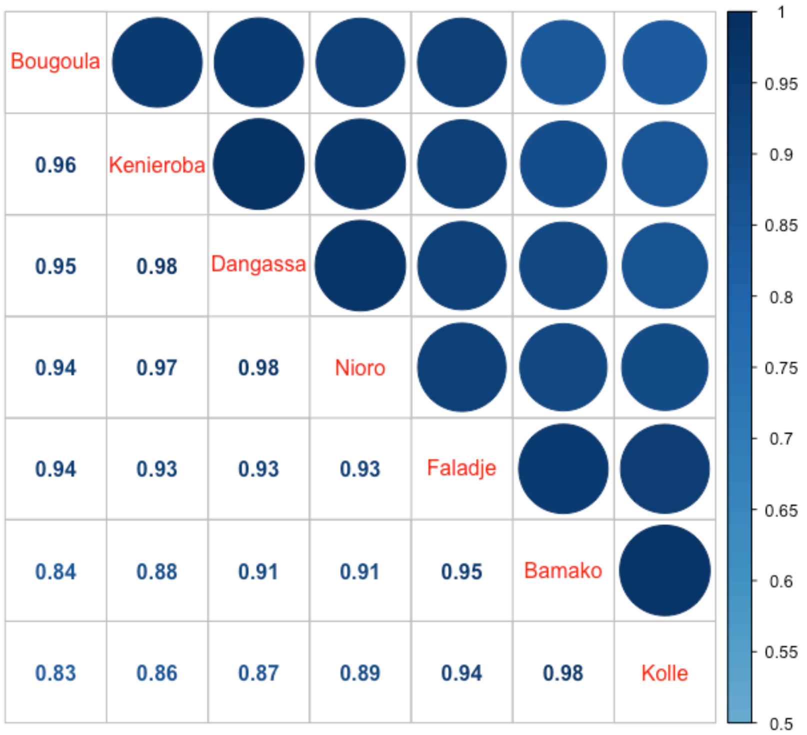

Figure S6. Correlation in allele frequencies across SNPs in drug resistance genes between geographic populations sampled from 2007 to 2017.

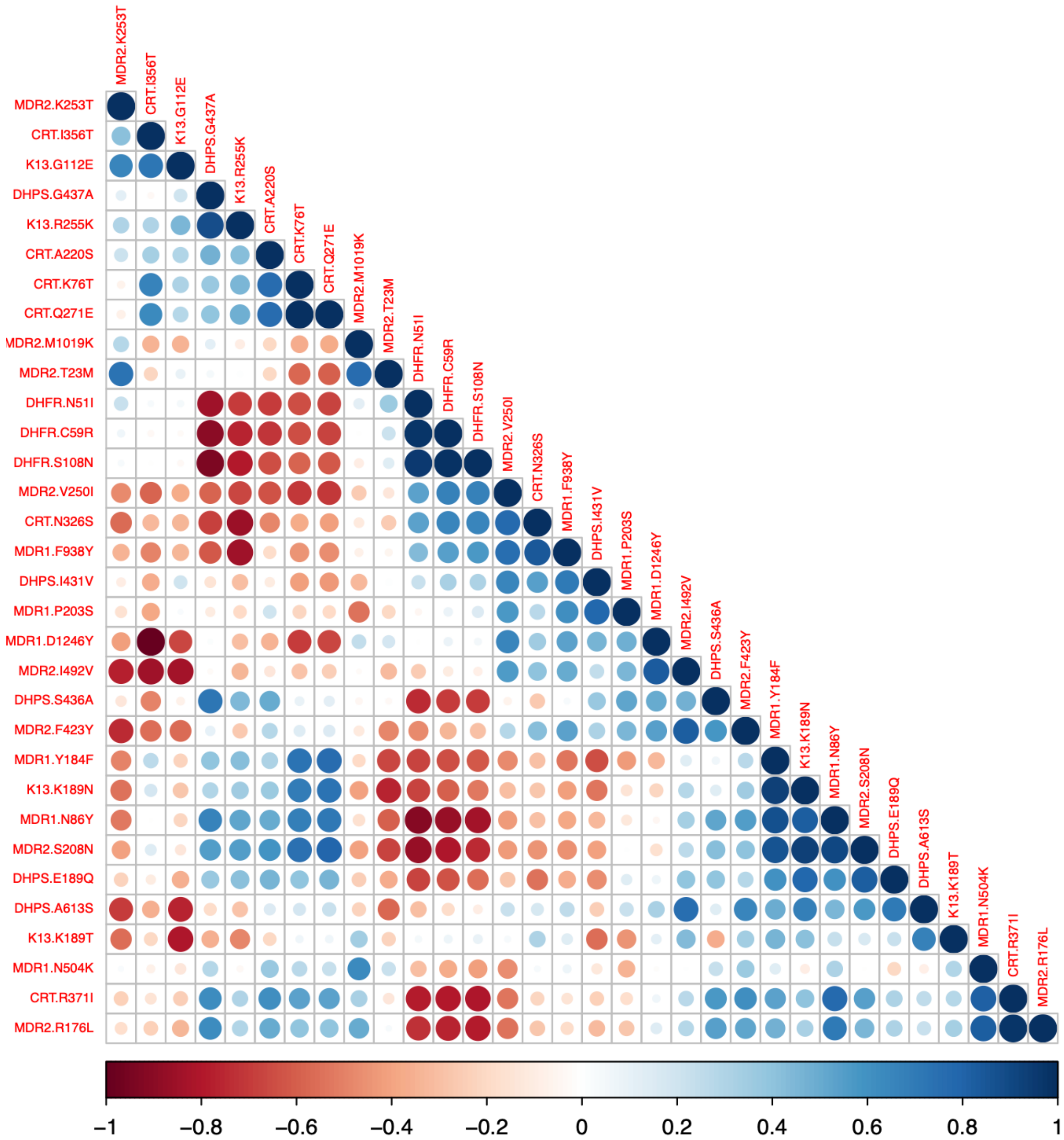

Figure S7. Correlation in overall allele frequencies between different SNPs in drugs resistance associate genes in *P. falciparum* isolates collected in Mali from 2007 to 2017 across different geographic sites.

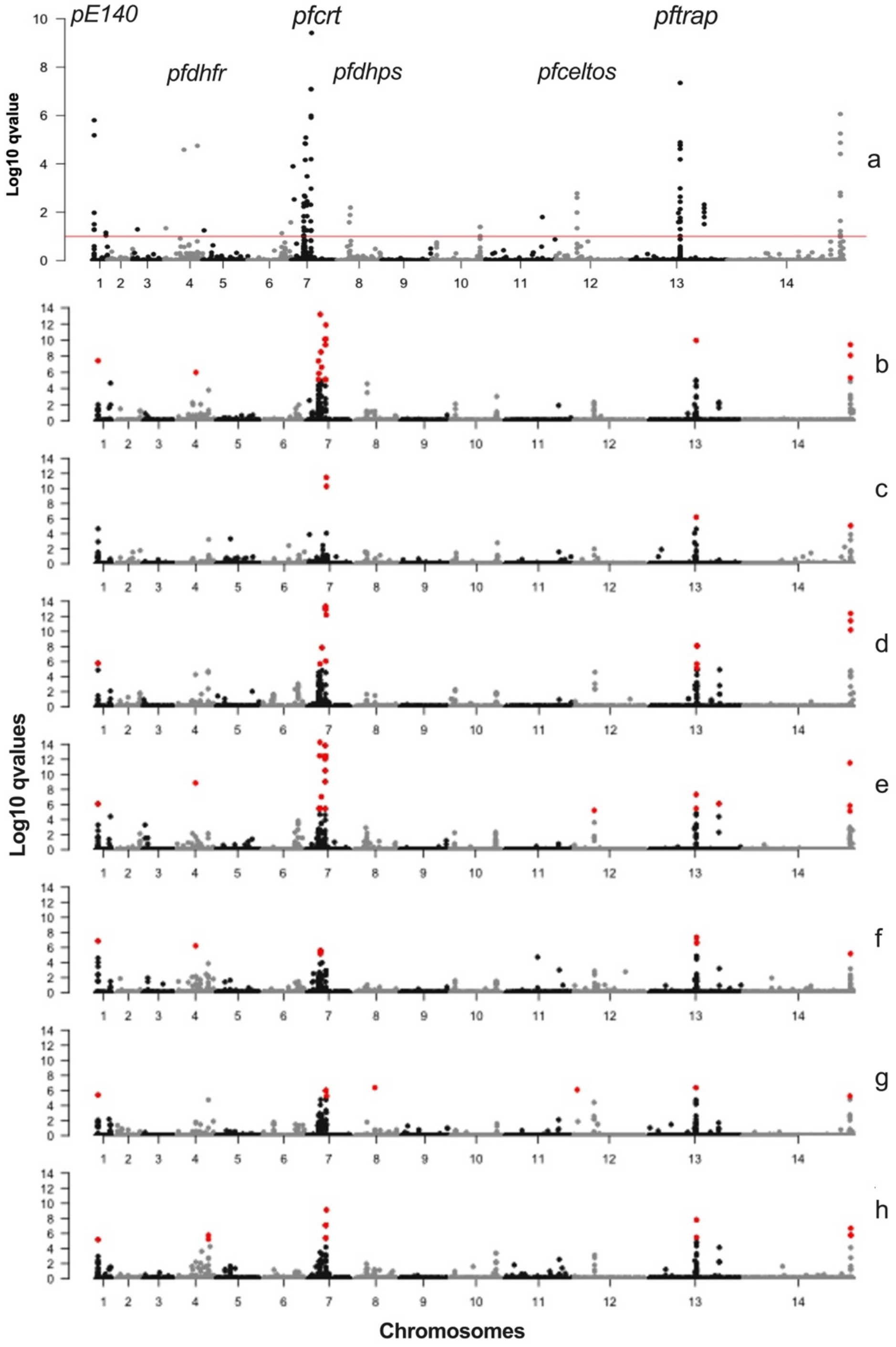

Figure S8. Manhattan plots of −log10 q-values (fdr adjusted p-values) of standardized integrated haplotype score (iHS) across the genome; a) all sampled geo-temporal populations in Mali b), c) , d) , e), f) , g) , h) . Each point shows a SNP on their respective chromosomes on the x-axis. Regions with SNP loci above a set significance threshold (redline) are candidate regions of selective sweeps.

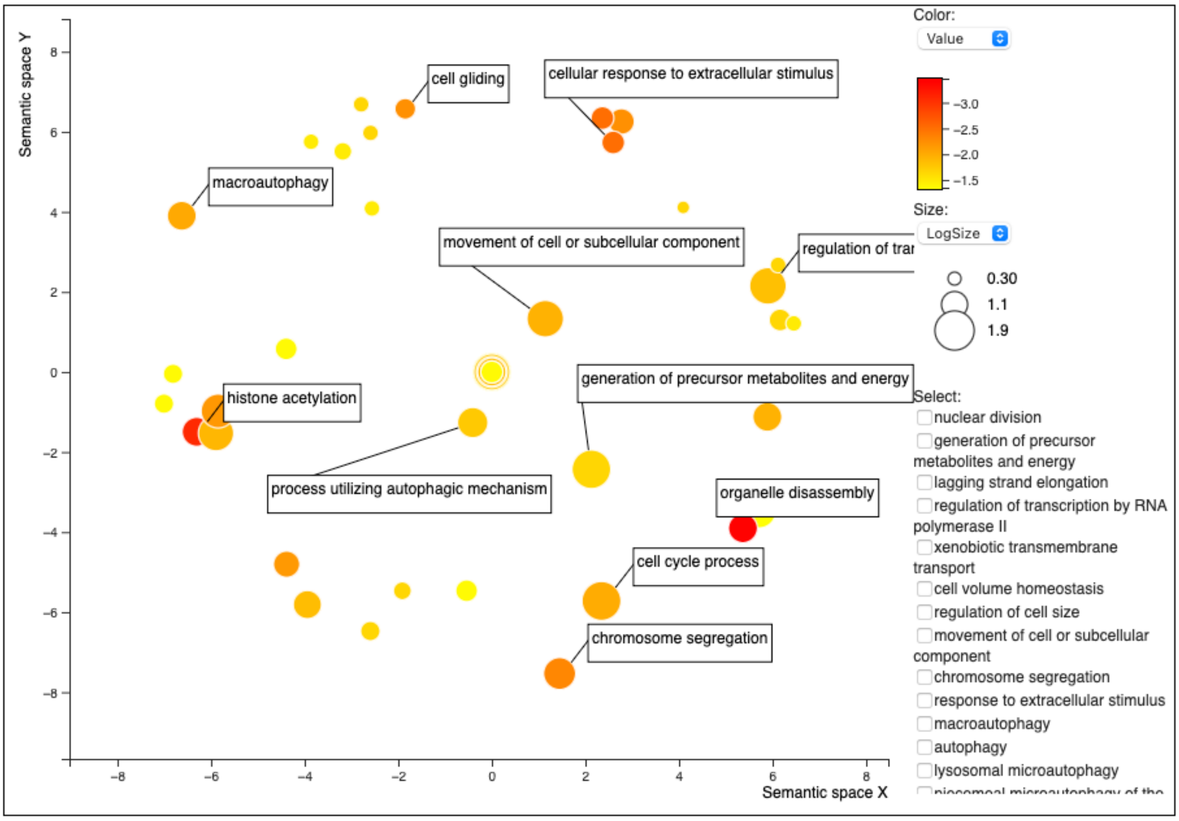

Figure S9. Cluster graph of gene ontologies for top genes with candidate signatures of selective sweeps from geo-temporal populations. Each point is shaded from yellow to red depending on the significance with the size of the points being proportionate to number of genes supporting the function.

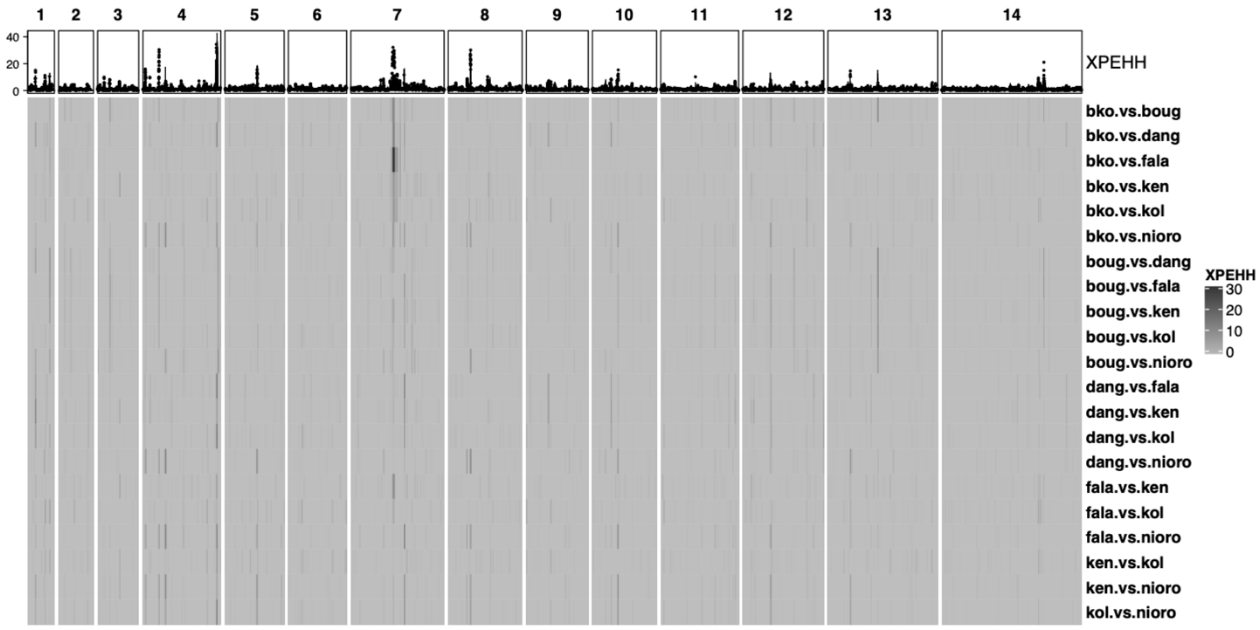

Figure S10. Heatmap of −log10 q-values (fdr corrected p-values) of the cross population extended haplotype homozygosity test (XP-EHH) indicating evidence of population specific difference in selection. The dark highlights represent strong evidence of extended haplotypes in one or other of the population. Each column represents a chromosome, and the rows show -log10 q-values of XP-EHH for pairs of geographic populations (bko=Bamako, boug=Bougoula-Hameau, dang=Dangassa, fala=Falaje, ken-kenieroba, kol=kolle, and nioro=Nioro).

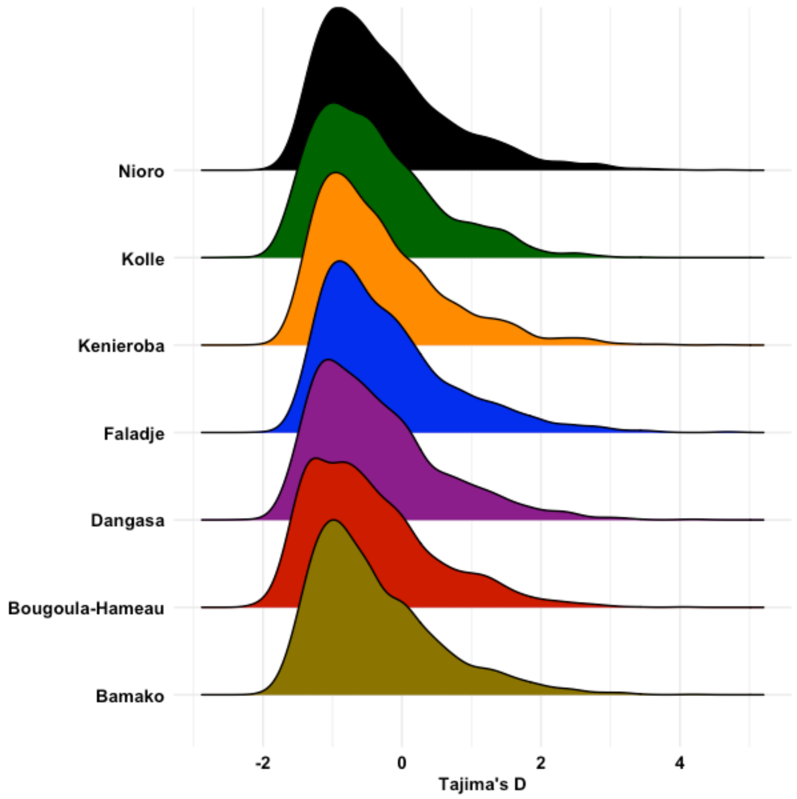

Figure S11. Tajima's D distributions across all populations. The distribution for each population is shown in rows as labelled on the y-axes.

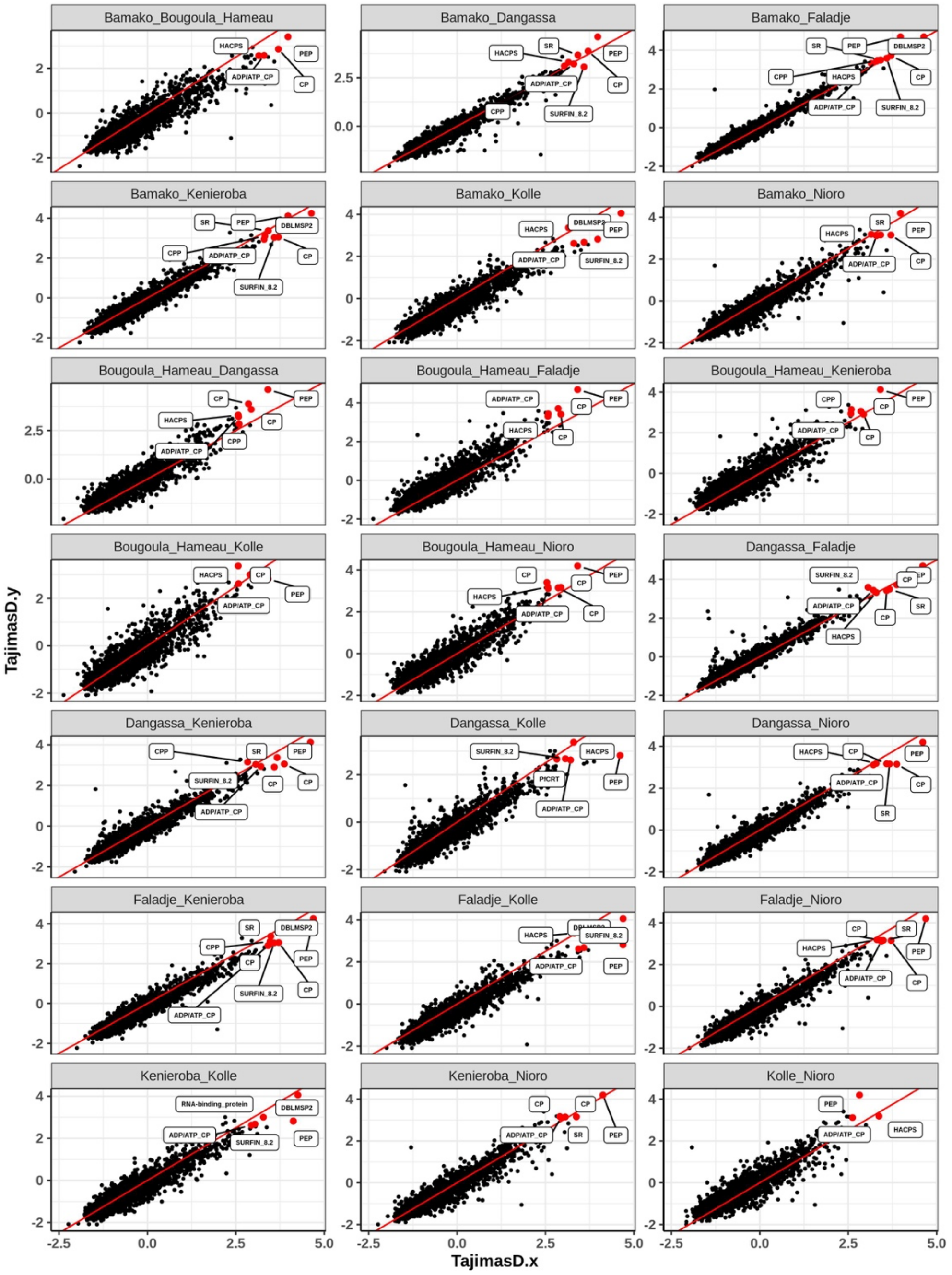

Figure S12. Pairwise correlation of Tajima's D values between populations. Top genes were selected and common genes between populations are highlighted above each plot labelled with the pair of populations being corelated. Unannotated protein groups include; CP (conserved proteins), CPP (Conserved Plasmodium Proteins), PEP (Plasmodium Exported proteins), SR(Surface related proteins). Known proteins include; Surface interspersed proteins (SURFIN8.2), Duffy binding Merozoite Surface Protein 2 (DBMSP2), ADP/ATP carrier proteins (ADP/ATP_SP), Holo-[acyl-carrier-protein] synthetase (HACPS) and Chloroquine resistance transporter (Pfcrt).
